# Transforming Prediction into Decision: Leveraging Transformer-Long Short-Term Memory Networks and Automatic Control for Enhanced Water Treatment Efficiency and Sustainability

**DOI:** 10.3390/s25061652

**Published:** 2025-03-07

**Authors:** Cheng Qiu, Qingchuan Li, Jiang Jing, Ningbo Tan, Jieping Wu, Mingxi Wang, Qianglin Li

**Affiliations:** 1 Department of Material and Environmental Engineering, Chengdu Technological University, Chengdu 611730, China; innovationcdtu@126.com (C.Q.); dachuandd@gmail.com (Q.L.);; 2 Sichuan Engineering Research Center for Small & Medium-Sized Intelligent Sewage Treatment Equipment, Chengdu 611730, China; 3 School of Chemical and Environmental Engineering, Wuhan Institute of Technology, Wuhan 430070, China

**Keywords:** ammonia nitrogen prediction, sequencing batch reactor, transformer network, long short-term memory network, automatic control

## Abstract

The study addresses the critical issue of accurately predicting ammonia nitrogen (NH_3_-N) concentration in a sequencing batch reactor (SBR) system, achieving reduced consumption through automatic control technology. NH_3_-N concentration serves as a key indicator of treatment efficiency and environmental impact; however, its complex dynamics and the scarcity of measurements pose significant challenges for accurate prediction. To tackle this problem, an innovative Transformer-long short-term memory (Transformer-LSTM) network model was proposed, which effectively integrates the strengths of both Transformer and LSTM architectures. The Transformer component excels at capturing long-range dependencies, while the LSTM component is adept at modeling sequential patterns. The innovation of the proposed methodology resides in the incorporation of dissolved oxygen (DO), electrical conductivity (EC), and oxidation-reduction potential (ORP) as input variables, along with their respective rate of change and cumulative value. This strategic selection of input features enhances the traditional utilization of water quality indicators and offers a more comprehensive dataset for prediction, ultimately improving model accuracy and reliability. Experimental validation on NH_3_-N datasets from the SBR system reveals that the proposed model significantly outperforms existing advanced methods in terms of root mean squared error (RMSE), mean absolute error (MAE), and coefficient of determination (R^2^). Furthermore, by integrating real-time sensor data with the Transformer-LSTM network and automatic control, substantial improvements in water treatment processes were achieved, resulting in a 26.9% reduction in energy or time consumption compared with traditional fixed processing cycles. This methodology provides an accurate and reliable tool for predicting NH_3_-N concentrations, contributing significantly to the sustainability of water treatment and ensuring compliance with emission standards.

## 1. Introduction

With the acceleration of industrialization and the continuous growth of the population, the problem of water pollution is becoming increasingly severe [1], and the demand for water treatment technology is also increasing [2]. Real-time water quality monitoring has emerged as a critical component in ensuring the safety and reliability of water supply systems. The significance of real-time monitoring lies in its ability to provide immediate insights into the composition and quality of water, enabling operators to make informed decisions that can significantly impact both public health and environmental sustainability [3]. Real-time data collection allows for the continuous assessment of various water quality parameters, such as ammonia and chemical oxygen demand. By implementing a proactive monitoring system, water treatment facilities can not only ensure compliance with regulatory standards but also build public trust in their ability to deliver safe water.

Moreover, adapting treatment strategies based on real-time water quality data is essential for optimizing operational efficiency and resource allocation [4]. For instance, if the monitoring system detects that contaminant levels have not significantly decreased, operators can respond by extending the treatment time to ensure the water meets safety standards before discharge. Conversely, if water quality meets specific criteria, early discharge can prevent the accumulation of treated water and minimize operational costs associated with storage, potentially leading to significant cost savings. Implementing such strategies not only enhances the overall efficiency of water treatment processes but also contributes to the sustainability of water resources.

Real-time monitoring of water quality parameters, such as NH_3_-N and other critical control indicators, often faces significant challenges, primarily due to the high costs and extensive workloads involved. However, soft sensing has emerged as a promising solution to address these issues. By leveraging easily accessible data, such as conductivity and redox potential, soft sensing allows for the indirect estimation of target water quality indicators through the development of predictive models [5]. This approach not only reduces operational costs but also increases efficiency, offering a cost-effective and scalable alternative for continuous water quality monitoring.

To further enhance the effectiveness of these adaptive strategies, mathematical methods are becoming increasingly relevant. By integrating real-time water quality data with sophisticated algorithms, operators can gain deeper insights into treatment processes and make more informed decisions.

Advanced computational methods, especially machine learning, and deep learning, are gradually emerging in water treatment engineering, providing new possibilities for predicting and optimizing treatment processes [6]. The advent of advanced computational methodologies has revolutionized the predictive analytics landscape in water treatment engineering [7], offering unprecedented insights into the dynamics of complex systems.

Methods (as shown in Table 1) have been proven effective in modeling the complex relationships between various factors and predicting quality indicators, which help to reduce the workload of environmental monitoring.

Although these methods have shown significant effectiveness in modeling complex relationships and predicting pollutant concentrations, they still face many challenges when dealing with data that has complex spatiotemporal dependencies [18]. Therefore, it is particularly important to explore more efficient and accurate prediction models [19].

The integration of sophisticated neural network architectures, including long short-term memory (LSTM) networks and Transformer models, has garnered significant attention in predictive modeling tasks [20]. The amalgamation of these architectures presents a formidable framework for temporal sequence analysis, capable of capturing intricate spatiotemporal dependencies inherent in the processes [21].

Given the advantages of LSTM in capturing long-term dependencies in time series data and the advantages of the Transformer in global context modeling [22], an innovative method, the Transformer-LSTM network, was proposed to improve prediction accuracy and generalization ability of NH_3_-N in SBR water treatment facilities.

The integration of LSTM networks, well-regarded for their proficiency in managing long-range dependencies in time series data, with the transformative capabilities of Transformer architectures, which excel at global context modeling, has opened new avenues for enhancing predictive analytics in complex systems. In particular, the proposed Transformer-LSTM network stands out as an innovative approach tailored to optimize the prediction accuracy and generalization ability of NH_3_-N levels in SBR water treatment facilities. By leveraging the strengths of both models, the hybrid framework not only aims to refine real-time predictive performance but also aspires to address the challenges posed by the dynamic and often nonlinear nature of wastewater treatment processes.

While LSTM networks excel at capturing short-term temporal dependencies in time series data, their ability to model long-range dependencies can be limited. Conversely, Transformer networks are adept at capturing long-range dependencies through the self-attention mechanism but might struggle with the sequential nature of data inherent in SBR processes [23]. To address these limitations, this study proposes a novel hybrid architecture that combines the strengths of both models. The Transformer-LSTM network leverages the Transformer’s ability to capture global context and the LSTM’s proficiency in handling sequential patterns within the SBR’s cyclical operation [24].

The proposed architecture consists of a Transformer encoder followed by a LSTM decoder. The Transformer encoder processes the entire time series of input variables (e.g., EC, DO, ORP, and their variants) simultaneously, generating a contextualized representation that captures the overall system state. This representation serves as input to the LSTM decoder, which sequentially predicts the NH_3_-N concentration over time. This two-stage approach enables the model to effectively capture both long-range dependencies (via the Transformer) and the inherent sequential nature of the SBR process (via the LSTM).

The encoding layer, positioned before the Transformer encoder, provides positional information for the input data. This enables the model to learn both positional and feature information. The Transformer encoder layer employs a multi-head attention mechanism and a feed-forward network to process the data [25]. Layer normalization is applied after each component to enhance network stability and prevent issues like gradient explosion. Additionally, residual connections are incorporated to preserve original data features during processing.

The core innovation of the proposed model lies in the reconstruction of the Transformer’s decoder layer. Instead of using a linear layer, which can lead to significant deviations in short dataset predictions, the decoder is replaced with an LSTM-based structure. The LSTM decoder consists of a linear rectification layer, an LSTM operation layer, and a fully connected layer. The LSTM operation layer, comprising input, forget, and output gates, processes data sequentially, ensuring the retention of temporal features critical for accurate predictions.

The Transformer-LSTM network processes data in two stages: intra-unit and inter-unit processing. Intra-unit processing involves parallel data processing through the positional encoding and Transformer encoder layers, while inter-unit processing relies on the LSTM decoder for sequential data handling. This dual approach ensures the model retains both global and temporal features, enhancing its predictive performance.

**Figure 1:**
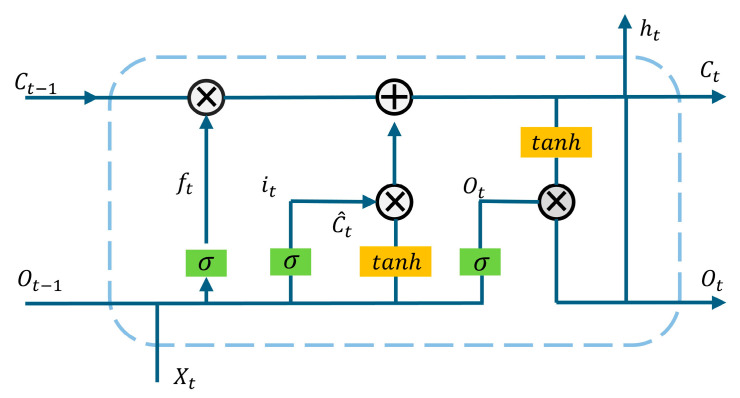
LSTM neural network structure.

**Figure 2:**
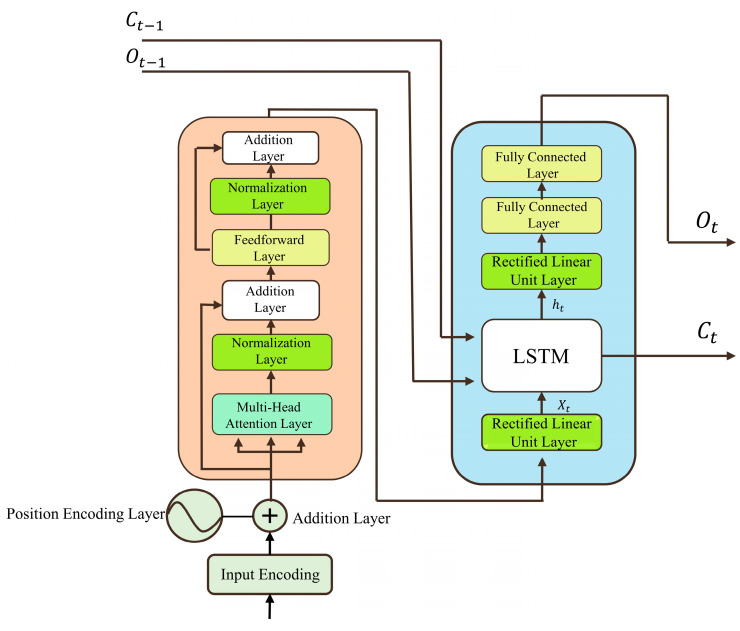
Transformer-LSTM inner-unit network structure.

**Figure 3:**
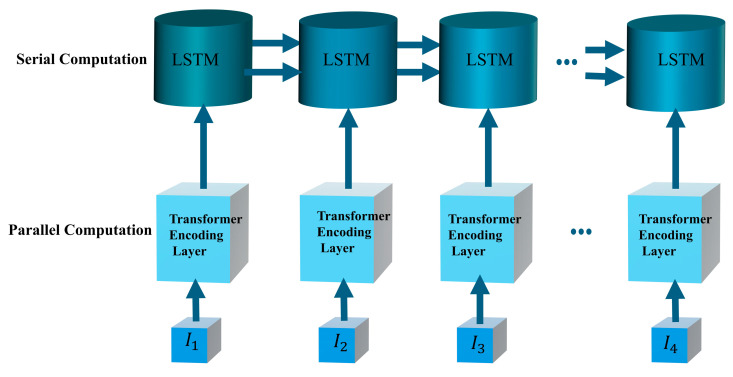
Transformer-LSTM outer-unit network structure.

The network structure is illustrated in Figure 1, Figure 2 and Figure 3, which depict the LSTM neural network, the inner-unit network structure, and the outer-unit network structure, respectively.

The primary objective of this investigation is to propel the frontier of predictive modeling and operational optimization in water treatment engineering by seamlessly integrating machine learning, specifically the Transformer-LSTM network, with automatic control systems. By harnessing real-time sensor data and leveraging the predictive capabilities of this advanced methodology, we endeavor to not only accurately forecast NH_3_-N concentrations in SBR systems but also to significantly enhance operational efficiency and reduce energy consumption. This holistic approach not only fosters regulatory compliance but also advances environmental stewardship within the water treatment industry, contributing significantly to a more sustainable future.

## 2. Methodology and Application

### 2.1. Structure of SBR

A sequencing batch reactor (SBR), receiving sewage water from a residential area, is prepared in this study. The capacity of the SBR, installation positions of inlet and outlet pumps, installation position of the mixing blade, location of aeration holes, as well as the source of wastewater, are detailed in this reference [26].

### 2.2. Control

**Figure 4:**
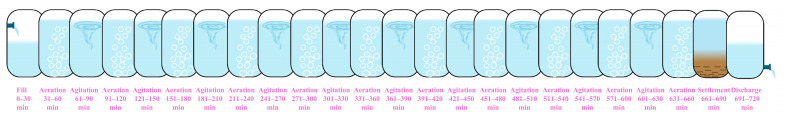
Treatment process of SBR.

The SBR process is automated and controlled by a PIC (programmable integrated circuit). The cycle, which lasts for 12 h, includes the following stages: 30 min fill-in stage, 630 min oxidation and agitation (alternating aeration and agitation, with aeration lasting for 30 min and agitation 30 min) stage, 30 min settlement stage, and 30 min discharge stage. Figure 4 shows the time management of the SBR operation.

### 2.3. Monitoring

Monitoring wastewater samples were taken from a collection vessel in the SBR tank, which allowed filtered water to pass through in order to eliminate the interference of activated sludge. Filtered EC, DO, and ORP were tested by monitoring sensors manufactured by Zhejiang Lohand Environment Technology Co., Ltd. of China (Hangzhou, China) (EC sensor LuHeng 665; DO sensor LuHeng 229; ORP sensor LuHeng 402).

Filtered NH_3_-N was tested by Nessler’s reagent colorimetry method (SP-756P UV visible photometer of Spectrum instruments of Shanghai (Shanghai, China)), filtered electronic conductivity and temperature were measured at 1 min intervals by sensors, while NH_3_-N was measured at 10 min intervals. In order to maintain consistency with the frequency of conductivity and temperature measurements and to ensure sufficient sample size for modeling, the unmeasured ammonia nitrogen values were filled in using linear interpolation. The filled ammonia nitrogen values were consistent with the number of temperature and conductivity values, i.e., one value per minute.

**Figure 5:**
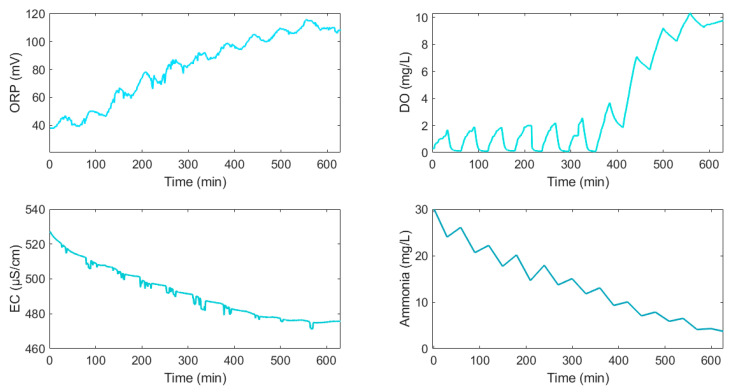
Typical variation of NH_3_-N, EC, DO, and ORP during a fixed process cycle.

Sensors were fitted approximately 200 mm below the liquid level within the reaction tank and above any potential sludge blanket that might be formed during settlement. All instruments were calibrated, maintained, and operated in accordance with the manufacturers’ instructions. During a process cycle, oxidation-reduction potential, dissolved oxygen, conductivity, and ammonia exhibited a certain pattern of change, as illustrated in Figure 5.

### 2.4. Assessed Input Variables

**Figure 6:**
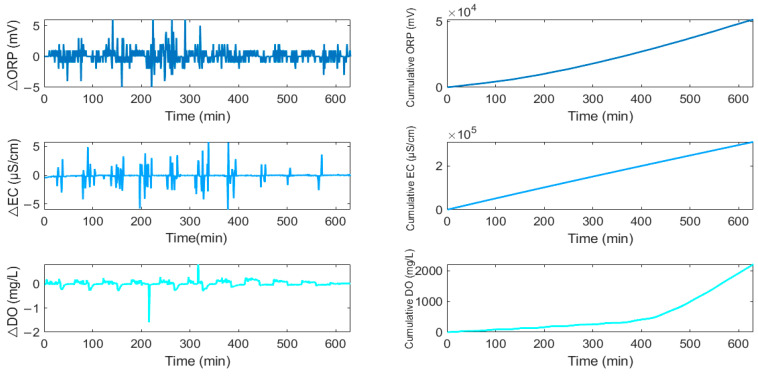
Typical processed input variables of EC, DO, and ORP during a fixed process cycle.

A series of unprocessed and processed input variables (Table 2), including raw EC, DO, and ORP, as well as their expanded value, were constructed and included in the set of independent variables. Processed input variables are illustrated in Figure 6.

The innovation of the proposed methodology lies in the incorporation of DO, EC, and ORP as input variables, along with their respective rates of change and cumulative values, to develop a predictive model for NH_3_-N. This strategic selection of input features enhances the traditional utilization of water quality indicators and offers a more comprehensive dataset for NH_3_-N prediction, ultimately facilitating improved model accuracy and reliability [27].

Each variable set included a unique collection of input variables. Within each 720 min cycle, data collected from 0 to 30 min and 661 to 720 min were excluded to eliminate the effects of filling and settlement periods (as these phases were not part of the biological reaction phases of the treatment cycle).

Data from 40 treatment cycles were collected, 12 (30%) of which were randomly separated for use as a test dataset, and the remainder were used as a training dataset.

### 2.5. Model Training and Evaluation

A Transformer-LSTM network model is implemented to predict NH_3_-N concentration in the water treatment process of a sequencing batch reactor based on the aforementioned input variables and data sets.

The proposed Transformer-LSTM network architecture is specifically designed to effectively capture both long-range dependencies and the sequential characteristics of the sewage treatment process [22]. This hybrid approach leverages the strengths of both the Transformer and LSTM models, aiming to enhance predictive performance beyond what either model can achieve individually.

The architectural rationale includes the following key components:

Transformer Encoder: The Transformer encoder processes the complete time series of input variables simultaneously. This capability allows the model to capture global context and long-range dependencies using the Transformer’s self-attention mechanism, which excels in understanding complex relationships across the entire dataset [28].

LSTM Decoder: The LSTM decoder is employed to predict NH_3_-N concentration over time in a sequential manner. LSTM networks are particularly adept at managing the inherent sequential patterns and cyclical behaviors present in sewage treatment processes. This sequential processing is crucial, as it enables the model to effectively learn from historical data and make accurate future predictions, something that can be challenging for the Transformer alone [29].

By combining the Transformer encoder with the LSTM decoder in a cascaded architecture, the model can leverage the complementary strengths of each component. The Transformer encoder focuses on extracting high-level contextual representations while the LSTM decoder processes these representations in a temporal sequence to generate final predictions.

To enhance the architecture further, several design choices have been implemented:

Positional Encoding Layer: This layer incorporates temporal position information into the input data, allowing the model to learn positional features alongside the data features, which is crucial for understanding the sequence order.

Multi-Head Attention: The multi-head attention mechanism within the Transformer encoder facilitates the model’s ability to concurrently attend to various information from different representation subspaces, thereby improving its feature extraction capabilities.

Custom LSTM Decoder: The traditional Transformer decoder has been replaced with a specially designed LSTM decoder layer. This modification is advantageous for time series prediction tasks, as LSTMs are inherently more suited to handling the sequential interdependencies of such data than a standard linear layer.

The model is trained using the Adam optimizer, with a learning rate of 0.001 and a loss function based on mean squared error (MSE). The model is trained for a total of 100 epochs, and the best model is selected based on the lowest validation loss.

For the LSTM decoder layer, the number of LSTM units was set to 32, and a dropout rate of 0.2 was applied to mitigate overfitting. The LSTM was equipped with forget gates to regulate the flow of information through time.

The Transformer encoder layer consisted of multiple attention heads, with the number of heads set to 4. Positional encoding was utilized to provide the model with awareness of the temporal order of the input sequence. The parameters of the proposed model are shown in Table 3.

The effectiveness of the machine learning models was evaluated across three criteria [30], which are R^2^, RMSE, and MAE.

## 3. Results and Analysis

### 3.1. Comparison of Models

**Figure 7:**
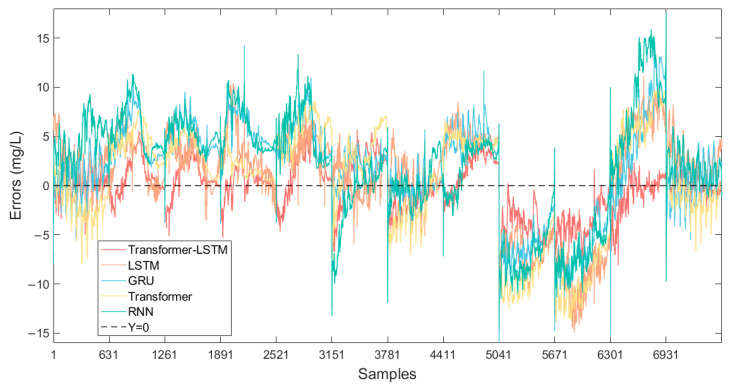
Prediction errors of all methods.

Transformer-LSTM as well as other models are utilized to train and predict NH_3_-N based on the air dataset. Table 4 compares the performance of different models on key evaluation metrics such as R^2^, RMSE, and MAE on the test set, while Figure 7 illustrates the prediction errors (predicted value minus measured value) of the models.

**Figure 8:**
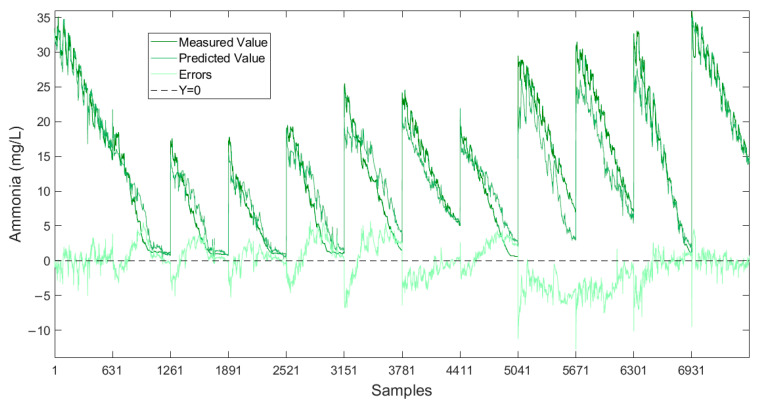
Prediction results of the Transformer-LSTM method.

By utilizing different neural networks to predict NH_3_-N, the results indicate that the Transformer-LSTM method achieved better performance on the test set, manifested by a R^2^ of 0.9255, as well as a lower RMSE of 2.6306 and a MAE of 2.0430. Figure 8 shows the prediction results of the Transformer-LSTM method applied to the test set. This suggests that the proposed methodology can significantly improve predictive performance, potentially capitalizing on the complementary advantages between different models, thereby enhancing overall predictive efficacy.

Integrated Transformer and LSTM models present a compelling approach to enhance predictive accuracy and stability for several reasons:

(1) Model Synergy: While the Transformer focuses on parallel data processing, attending to specific data parts to ensure feature retention and enhance prediction accuracy [31], LSTM handles sequential data processing, capturing overall data trends to ensure sufficient feature coverage and improve prediction precision. By combining these models, their complementary strengths synergistically collaborate, particularly beneficial for tasks requiring simultaneous consideration of sequence and context comprehension [32].
(2) Fusion for comprehensive model amalgamation: In the realm of model integration, the fusion technique provides a robust alternative. Here, the predictions from the Transformer and LSTM are amalgamated using a fusion mechanism, such as weighted averaging or feature concatenation. This fusion process capitalizes on the strengths of both models, ensuring a comprehensive representation of the data for enhanced predictive accuracy [33]. The adaptability of fusion techniques allows for nuanced integration, potentially outperforming conventional ensemble methods [34].
(3) Mitigating Overfitting: The ensemble of Transformer and LSTM models mitigates overfitting risks by leveraging diverse information sources and their complementarity. This fusion method enhances model generalization, enabling more accurate predictions of unseen data. By integrating global and sequential information, stacked models achieve robust performance across various tasks and datasets.

### 3.2. Processes Optimization by the Transformer-LSTM Network

The ability to predict ammonia levels in wastewater through machine learning models holds immense potential for reducing the workload associated with traditional water quality monitoring methods, thus enabling more efficient and targeted water quality management.

**Figure 9:**
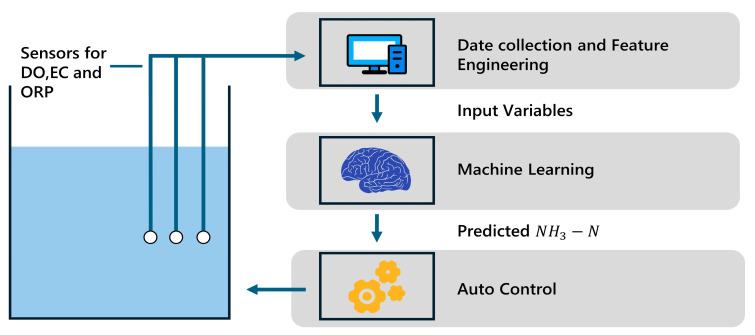
The technical balance between the SBR and machine learning.

Moreover, the integration of machine learning with automation technologies in wastewater treatment plants can further enhance the efficiency and sustainability of the treatment process [35]. By utilizing sensors and actuators, the treatment plant can automatically adjust its operational parameters based on the predicted ammonia nitrogen levels. This flexibility in the treatment scheme allows for more efficient resource utilization, reducing energy consumption and optimizing the use of chemicals and other treatment agents. Figure 9 illustrates the technical balance between the SBR and machine learning. This figure serves as a visual representation of the interplay and relationship between these two components in a wastewater treatment system.

In the new 20 cycles of water treatment (a completely separate set of data that was collected after the initial 40 cycles), real-time dissolved oxygen (DO), electrical conductivity (EC), and oxidation-reduction potential (ORP) were collected by sensors and input into the established machine learning model (utilizing the proposed Transformer-LSTM methodology) to obtain the output value of ammonia nitrogen. An automatic operation plan for the sequencing batch reactor treatment system was set up, where the biochemical reaction would be halted immediately and enter the sedimentation and drainage phase when the output value of ammonia nitrogen was lower than the current national emission standard of China (NH_3_-N ≤ 5 mg/L).

### 3.3. Circuit Design for Automated SBR

(1) Sensors

The LuHeng sensors (DO Sensor 229, EC Sensor 665, and ORP Sensor 402) provide continuous analog signals that represent the dissolved oxygen levels, electrical conductivity levels, and oxidation-reduction potential levels in the SBR tank, respectively.

(2) Data Acquisition Module

This module interfaces with the sensors, converting analog signals into digital data. It uses an analog-to-digital converter with appropriate input ranges for each sensor. The data acquisition module also incorporates a microcontroller to handle data sampling, timing, and initial processing. The sampling rate is at least 1 min. The microcontroller sends the digitized sensor data to the next stage.

(3) Transformer-LSTM Model

This model resides on a computer with sufficient processing power. The data acquisition module transmits the sensor data to the computer via a communication link. The Transformer-LSTM model receives this data, performs its prediction of NH_3_-N concentration, and outputs the predicted concentration value.

(4) Control Algorithm

This algorithm runs on the computer alongside the Transformer-LSTM model. It continuously receives the predicted NH_3_-N level from the model. The core logic is as follows: if the predicted NH_3_-N is ≤5 mg/L, initiate the stop and discharge sequence. This algorithm needs to manage the timing to ensure the PropMiner sequence transitions within the SBR cycle.

(5) Actuators/Execution

The control algorithm sends commands to the actuators. The actuators would control the various stages of the SBR process (fill, oxidation/agitation, settlement, discharge).

(6) Communication

A robust communication network would be crucial to connect all the components. This automated circuit design scheme is illustrated in detail in Appendix A. Meanwhile, the ammonia nitrogen in the water sample at this point (point of discharge) was manually measured.

Experimental results demonstrate the predicted values of ammonia nitrogen during the biochemical reaction phase of the SBR system and the actual values of ammonia nitrogen measured at the point of discharge.

The data collected from the sensors revealed a close correlation between the real-time measurements of DO, EC, and ORP and the output value of ammonia nitrogen predicted by the machine learning model. As the SBR system progressed through its cycles, the model was able to accurately predict the ammonia nitrogen concentration in the water.

**Figure 10:**
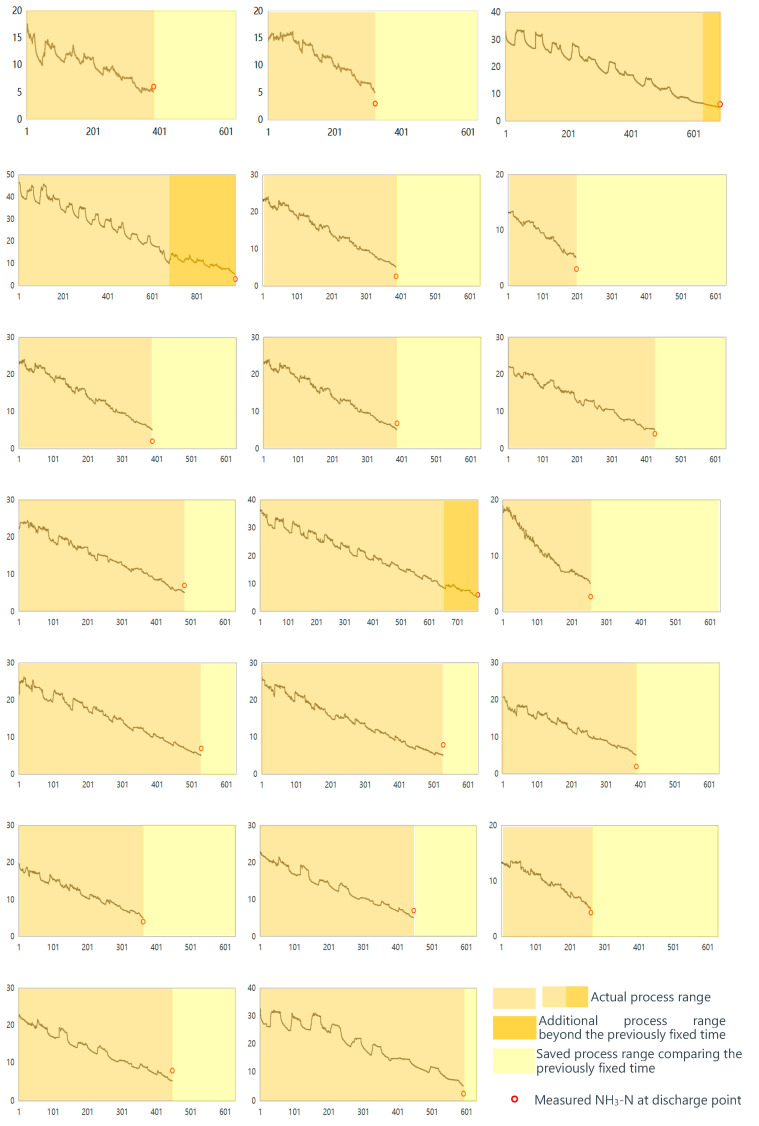
Predicted NH_3_-N by Transformer-LSTM network and process range on 20 cycles (All horizontal axes represent time (min), and all vertical axes represent NH_3_-N (mg/L).

At the same time, water samples were manually collected from the system at the point of discharge. These samples were then analyzed to determine the actual concentration of ammonia nitrogen. The results (Figure 10) showed a high degree of accuracy between the predicted values from the machine learning model and the actual measured values at the discharge point.

The integration of machine learning with automatic control has demonstrated significant improvements in the new 20 cycles. Specifically, this approach has been found to save an impressive 26.9% of processing time or energy consumption compared with the traditional fixed process cycle utilized previously. The 26.9% improvement in energy or time consumption was calculated by comparing the average biochemical reaction time in the additional 20 cycles using the proposed method (460.35 min) with the fixed biochemical reaction time (630 min) from traditional operations. This represents a 26.9% reduction in time, which directly translates to proportional energy savings under the assumption that time equates to energy consumption in the SBR system. Detailed operation times for each of the additional 20 cycles are provided in Table 5 to further support this conclusion. Although machine learning models may not achieve 100% accuracy in predicting ammonia nitrogen concentrations, the observed error levels at the discharge point are deemed acceptable. The integration of these models with real-time sensor data offers significant benefits in optimizing water treatment processes.

These findings highlight the benefits of using machine learning models and real-time sensor data to optimize water treatment processes. By accurately predicting ammonia nitrogen concentrations and automatically adjusting the operation of the SBR system, it is possible to ensure that treated water meets environmental standards while minimizing the use of resources and energy.

## 4. Conclusions

This study demonstrates that integrating Transformer-LSTM networks with automated control systems significantly improves water treatment efficiency and operational sustainability. Through rigorous experimentation and analysis, three key findings emerge:

Superior Predictive Performance: The proposed Transformer-LSTM model achieved satisfactory results in prediction accuracy, with a R^2^ of 0.9255, as well as a lower RMSE of 2.6306 and a MAE of 2.0430. This enables reliable forecasting of ammonia nitrogen variations during SBR cycles, particularly at critical discharge points.

Operational Efficiency Gains: By coupling real-time sensor data with automated control logic, the system reduced energy/time consumption by 26.9% versus fixed-cycle operations. Dynamic adjustments to aeration and mixing duration based on predicted water quality parameters proved critical to these savings.

Practical Implementation Framework: The closed-loop control architecture successfully translated predictions into actionable adjustments, maintaining effluent ammonia levels below 5.0 mg/L while minimizing resource use. Field tests at 20 new SBR run cycles, excluding the training and testing sets, demonstrated a 26.9% cost reduction.

These results address two persistent challenges in water treatment: (1) The latency in manual quality monitoring. (2) The inflexibility of predetermined process parameters. While the current framework focuses on SBR systems, its modular design allows adaptation to other treatment modalities like anaerobic-anoxic-oxic. Future work will explore hybrid models incorporating spectral water quality data and expand validation to multi-plant scenarios. We also plan to conduct a comprehensive investigation into hyperparameter optimization for the Transformer-LSTM network architecture, employing methodologies such as grid search, random search, and Bayesian optimization. This systematic exploration will aim to refine the model further and provide deeper insights into the influence of specific parameter settings, such as the number of layers in the Transformer, the number of attention heads, and the number of hidden units in the LSTM, on model performance. By doing so, we hope to enhance the predictive accuracy and generalization capability of our approach, ultimately contributing to more effective and efficient wastewater treatment processes.

## Figures and Tables

**Table 1:** Machine learning methods used in the prediction of water/air quality indicators.

References	Variables/Inputs	Targets/Outputs	Model Performance	Model
[8]	BOD, DO, NH_3_-N et al.	Fault detection of urban wastewater treatment	Accuracy 99.7%	Transformer-LSTM
[9]	Readily biodegradable substrate, particulate inert organic matter, slowly biodegradable substrate, total suspended solids, flow rate, active autotrophic biomass et al.	Ammonia-nitrogen concentration in wastewater treatment processes	RMSE 3.76, MAE 2.96, R^2^ 80.94%	LSTM
[10]	Flow, velocity, liquid level, pH, conductivity	COD, BOD_5_, TP, TN, NH_3_–N in urban drainage	R^2^ 0.961, 0.9384, 0.9575, 0.9441, 0.9502; RMSE 8.3112, 6.7795, 0.2691, 2.6239, 1.4894	LSTM
[11]	COD, ammonium nitrogen, total nitrogen, total phosphorus	Future changes in water quality index	Prediction accuracy 85.85%, 47.15%, 85.66%, 89.07%	LSTM
[12]	DO, pH, and NH_3_-N	Water pollution trends	RMSE of the RF-CEEMD-LSTM model is reduced by 62.6%, 39.9%, and 15.5% compared with those of the LSTM, RF-LSTM, and CEEMD-LSTM models	LSTM
[13]	pH, TP	TN of a river in the Beijing–Tianjin–Hebei region of China	RMSE 0.2093 MAE 0.1552 R^2^ 0.9552	LSTM
[14]	Temperature, DO, EC, COD, TN	TP concentrations at the inlet of Taihu Lake, China	R^2^ 0.37~0.87	Transformer-LSTM
[15]	Adsorption conditions, pyrolysis conditions, elemental composition, biochar’s physical properties	Heavy metal ions from wastewater	R^2^ 0.98 RMSE 0.296 MAE 0.145	Transformer
[16]	Meteorology, temporal trending variables road traffic, wildfire perimeter, wildfire intensity	Hourly PM_2.5_ concentrations in wildfire-prone areas	RMSE 6.92	Transformer
[17]	Dew point, temperature, pressure, combined wind direction, cumulated wind speed, cumulated hours of rain, cumulated hours of relative humidity	Hourly air PM_2.5_	MAE 9	Transformer

**Table 2:** Input variables.

Input Variables	Description
EC	Raw electronic conductivity data
ΔEC	ΔEC = EC_i_ − EC_i−1_, the difference between current value and previous
EC_Cum_	Cumulative EC value, EC_Cum_ = EC_1_ + EC_2_ + …+ EC_i_
DO	Raw dissolved oxygen data
ΔDO	ΔDO = DO_i_ − DO_i−1_, the difference between current value and previous
DO_Cum_	Cumulative DO value, DO_Cum_ = DO_1_ + DO_2_ + …+ DO_i_
ORP	Raw ORP data
ΔORP	ΔORP = ORP_i_ − ORP_i−1_, the difference between current value and previous
ORP_Cum_	Cumulative ORP value, ORP_Cum_ = ORP_1_ + ORP_2_ + …+ ORP_i_

**Table 3:** Summary of the Transformer-LSTM Model Parameters.

Model Component	Parameter	Setting	Description
Positional Encoding Layer	Dimension	16	Ensures the model can effectively learn the position information of the data.
	Maximum Time Steps	30	Handles the longest input sequence.
Transformer Encoder Layer	Number of Attention Heads	4	Enhances feature extraction capabilities.
	Hidden Units per Layer	32	Ensures the model has sufficient expressiveness.
	Number of Layers	3	Enhances model depth to capture complex patterns.
LSTM Decoder Layer	LSTM Units	32	Maintains consistency with the Transformer encoder complexity.
	Dropout Rate	0.2	Mitigates overfitting.
Training Parameters	Optimizer	Adam	Uses the Adam optimizer.
	Learning Rate	0.001	Learning rate for the Adam optimizer.
	Loss Function	MSE	Based on Mean Squared Error (MSE).
	Training Epochs	100	Total number of training epochs.

**Table 4:** The performance of models on the test set.

Model	R^2^	RMSE	MAE	Hardware Requirements	Training Time (min)
Transformer-LSTM	0.9255	2.6306	2.0430	High-end GPU, 16 GB RAM	8
LSTM	0.8433	4.9433	4.0442	Standard CPU, 8 GB RAM	5
GRU	0.8367	5.0476	4.2178	Standard CPU, 8 GB RAM	5
Transformer	0.7549	5.4464	4.5084	High-end GPU, 16 GB RAM	8
RNN	0.7667	5.8387	4.8763	Standard CPU, 8 GB RAM	5

**Table 5:** Operation time by proposed methodology for additional cycles 41–60.

Cycle No.	41	42	43	44	45	46	47	48	49	50
Operation time by proposed methodology (mins)	393	312	662	994	390	198	388	382	420	484
Cycle No.	51	52	53	54	55	56	57	58	59	60
Operation time by proposed methodology (mins)	790	254	525	513	386	365	448	266	430	593

## Data Availability

Data will be made available on request.
